# Persistence of Crimean-Congo Hemorrhagic Fever Virus RNA

**DOI:** 10.3201/eid2602.191460

**Published:** 2020-02

**Authors:** Leholonolo Mathengtheng, Dominique Goedhals, Phillip A. Bester, Jacqueline Goedhals, Felicity J. Burt

**Affiliations:** National Health Laboratory Service, Bloemfontein, South Africa; University of the Free State, Bloemfontein

**Keywords:** Crimean-Congo hemorrhagic fever virus, CCHFV, RNA detection, viruses, orthonairoviruses, South Africa

## Abstract

Crimean-Congo hemorrhagic fever virus (CCHFV) causes severe disease with fatalities. Awareness of potential sources of infection is important to reduce risk to healthcare workers and contacts. We detected CCHFV RNA in formalin-fixed, paraffin-embedded tissues from a spontaneous abortion that were submitted for histology 9 weeks after a suspected CCHFV infection in the mother.

Crimean-Congo hemorrhagic fever virus (CCHFV) has the potential to emerge in areas where competent vectors are present and become a major risk to public health. Because of an absence of registered vaccines or specific antiviral treatment, CCHFV is included as one of the diseases prioritized by the World Health Organization for research and development in public health emergency contexts. Nosocomial infections are one of its transmission routes; thus, awareness of possible sources of infection is important for reducing risk to healthcare workers and other contacts of infected persons.

We detected a case of CCHFV infection in South Africa during a retrospective study, conducted in 2014, of serum samples from patients with suspected tickbite fever and no diagnosis. We retrospectively screened 196 serum samples that were collected during 2008–2011 from acutely ill patients in Free State Province and submitted to the routine serology laboratory of the Department of Medical Microbiology, National Health Laboratory Service (Bloemfontein, South Africa) with suspected rickettsial infection. The University of the Free State Health Science Ethics Committee provided ethics approval to screen residual diagnostic samples (ethics approval no. ETOVS 118/06). 

For this screening, RNA was extracted from human serum samples using the QIAamp Viral RNA Mini Kit (QIAGEN, https://www.qiagen.com), according to the manufacturer’s instructions. We used RNA as template in a nested reverse transcription PCR (RT-PCR) with CCHFV primers designated F2, R3 and F3, R2 (*1*,*2*). The primers target a 536-bp region (F2, R3) and a 260-bp region (F3, R2) of the small segment using a nested format. 

CCHFV RNA was amplified from 1 of the 196 samples. Determination of partial nucleotide sequence of the 260-bp amplicon confirmed that CCHFV RNA was amplified with 100% nt homology to previously identified South Africa strains (Figure). Because the screening was performed retrospectively on residual samples, no clinical details were available; however, a heat-inactivated aliquot of the serum had a CCHFV IgM titer of 1:40 and IgG titer of 1:10 by immunofluorescent antibody assays (EUROIMMUN, https://www.euroimmun.com), suggesting an acute infection. Laboratory records indicated that, 9 weeks after the blood sample was collected and submitted, an endometrial curettage sample taken after a spontaneous abortion was sent for histologic examination. Therefore, we retrieved a formalin-fixed paraffin-embedded tissue section of this sample from the archives and tested it retrospectively for evidence of CCHFV RNA using nested RT-PCR. We amplified a short fragment of the gene encoding the CCHFV nucleoprotein and confirmed its identity using nucleotide sequencing (Figure). 

**Figure:**
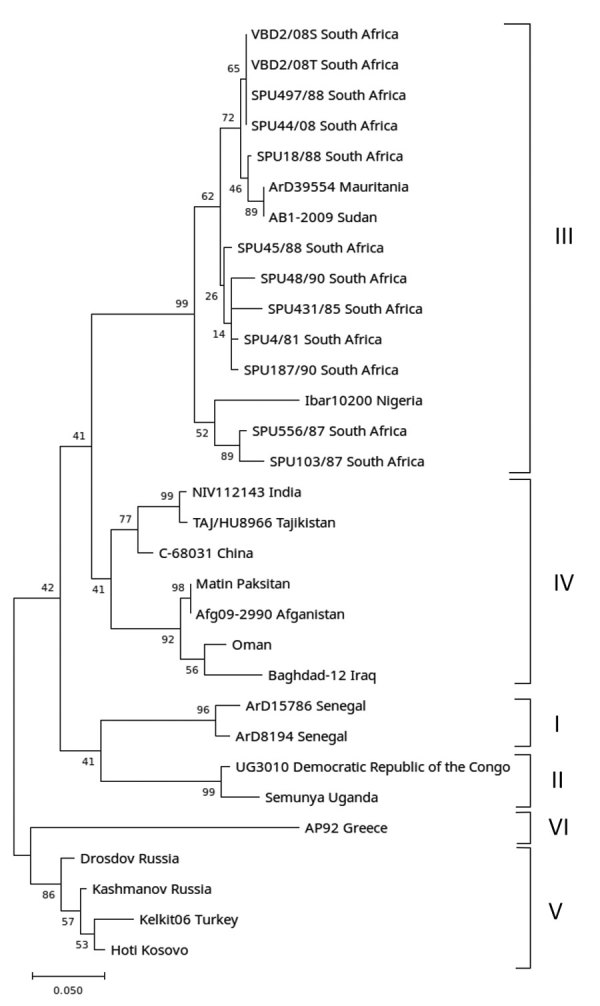
Detection of Crimean-Congo hemorrhagic fever virus (CCHFV) from a retrospectively tested human serum sample that was among those collected during 2008–2011 from acutely ill patients with suspected rickettsial infection, South Africa. Phylogenetic tree was constructed using a 186-bp region of the CCHFV small gene encoding the nucleoprotein sequence. Nucleotide data were obtained in the study for samples designated VBD2/08S (serum-derived), VBD 2/08T (tissue-derived), and retrieved from GenBank for 29 CCHFV isolates from similar, or geographically distinct, regions (GenBank accession number available on request). The tree was constructed using MEGA X (https://www.megasoftware.net) and 1,000 bootstraps. Nodes with values <50 were omitted from the figure. Branch labels are name of isolate and country of origin. Genotypes are indicated at right. Scale bar indicates nucleotide substitutions per site.

Because the biopsy sample contained both endometrial and placental tissue, the sample did not enable cellular localization of viral RNA or antigen and thus did not provide evidence directly linking the viral RNA to fetal demise. Similarly, the absence of retrospective maternal serum samples collected at the time of the spontaneous abortion, or fresh biopsy material, did not enable investigation for viremia. However, detection of viral RNA in tissue biopsy collected 9 weeks after detection of viral RNA in a serum sample suggests persistence of CCHFV RNA.

Persistent viral infection in selected sites has been demonstrated for other RNA viruses. Zika virus RNA is normally detectable in serum samples 3–10 days after symptom onset. Investigations on persistence of Zika virus RNA in formalin-fixed, paraffin-embedded fetal tissue from pregnant mothers with confirmed infection identified a time frame of 119–238 (mean 163) days from maternal symptom onset to detection of RNA by RT-PCR in brains and 15–210 (mean 81) days in placentas (*3*). The detection of viral RNA in a curettage specimen does not enable distinction between maternal and fetal infection but nevertheless extends the period during which viral RNA is detectable.

Little is known about the clinical course of CCHFV infections in pregnant women. Spontaneous abortions were reported in 24 (58.5%) of 41 mothers with CCHFV infection (*4*). Similarly, viral hemorrhagic fevers, such as from Ebola and Lassa viruses, during pregnancy have been associated with spontaneous abortion (*5*,*6*). Antigenemia has been detected 2–11 days after illness onset, viral RNA has been detected 1–18 days after initial symptoms, and infectious virus has been isolated from samples collected 1–12 days after onset (*7*). Whether pregnancy influences the duration of viremia is not known. 

Although our findings are subject to some limitations and it is not possible to make any assumptions with regard to association between virus infection and fetal demise or distinguish between maternal and fetal infection, our findings extend the period for detecting CCHFV RNA after infection and raises the the potential for nosocomial infections. In addition, because the serum sample we tested was initially submitted for suspected tickbite fever, healthcare workers should consider CCHFV as part of the differential diagnosis for tickbite fever (rickettsial infections) in local patients and travelers returning from CCHFV-endemic regions.
